# Metabolomics dataset of PPAR-pan treated rat liver

**DOI:** 10.1016/j.dib.2016.05.002

**Published:** 2016-05-10

**Authors:** Zsuzsanna Ament, James A. West, Elizabeth Stanley, Xuefei Li, Tom Ashmore, Lee D. Roberts, Jayne Wright, Andrew W. Nicholls, Julian L. Griffin

**Affiliations:** a Medical Research Council, Human Nutrition Research, Elsie Widdowson Laboratory, 120 Fulbourn Road, Cambridge CB1 9NL, UK; b The Department of Biochemistry, University of Cambridge, 80 Tennis Court Road, Cambridge CB2 1GA, UK; c The Cambridge Systems Biology Centre (CSBC), University of Cambridge, Cambridge CB2 1QR, UK; d Jayne Wright Ltd., Underhill House, Ledbury, Herefordshire HR8 2QR, UK; e GlaxoSmithKline, Investigative Preclinical Toxicology, Park Road, Ware, SG12 0DP, UK

**Keywords:** Peroxisome proliferator activated receptors, PPAR, Metabolomics, Lipidomics, Mass spectrometry

## Abstract

This article contains mass spectrometry (MS) data investigating small molecule changes as an effect of a triple peroxisome proliferator-activated receptor (PPAR-pan) agonist GW625019 in the liver as described in the manuscript (Ament et al., 2016) [1]. Samples were measured using gas chromatography-mass spectrometry (GC–MS) for total fatty acid content, and liquid chromatography-mass spectrometry (LC–MS) to measure intact lipids, carnitines and selected aqueous metabolites and eicosanoids. Data files comprise of Excel (Microsoft, WA, USA) spreadsheets of identified metabolites and their area ratio values for total fatty acids, carnitines, aqueous metabolites, and eicosanoids where the intensity of the analytes were normalised to the intensity of the internal standard. In the case of open profiling intact lipid data, the Excel file contains area ratio values of retention time and mass to charge ratio pairs; again, the area ratio values were calculated by normalising to the intensity of the internal standard. It should be noted that several metabolic changes are potentially indirect (secondary, tertiary and ensuing changes).

**Specifications Table**

**Table:** 

Subject area	*Biochemistry*
More specific subject area	*Metabolomics*
Type of data	*Mass Spectrometry data summarized in Excel spreadsheets*
How data was acquired	*GC-MS of total fatty acids: Trace GC Ultra coupled to a DSQ II*
	*LC-MS of intact lipids: Waters Xevo G2 (Q-ToF) mass spectrometer coupled to an Acquity UPLC.*
	*LC-MS of acyl-carnitines: AB Sciex 5500 Qtrap mass spectrometer coupled to an Acquity UPLC.*
	*LC-MS of aqueous metabolites: AB Sciex 5500 Qtrap mass spectrometer coupled to a SIL20-A LC system*
	*LC-MS of eicosanoid metabolites: AB Sciex 4000 Qtrap mass spectrometer coupled to an Acquity UPLC.*
Data format	*Peak picked and normalised to relevant internal standard and wet tissue weight*
Experimental factors	*Metabolites were extracted from liver tissue samples using a modified Bligh and Dyer procedure using chloroform, methanol and water*
Experimental features	*Liver metabolite response to different doses of PPAR-pan agonist*
Data source location	*Cambridge, United Kingdom*
Data accessibility	*Data is within this article and for the carnitine and targeted aqueous metabolites the data is accessible through the MetaboLights repository under study numbers MTBLS278 (eicosanoid data) and MTBLS303 (carnitine and aqueous metabolites).*

**Value of the data**

• These data provide a broad survey of rat liver metabolite changes due to PPAR-pan agonist treatment.
• The different datasets can be used to explore challenges of data merging and integration across analysis platforms.

• The study has both a dose response and drug recovery aspect allowing others to model these types of data.

## 1 **Data**

This article contains mass spectrometry data of small molecules, including open profiling assays for total fatty acids (GC–MS) and intact lipids (LC–MS) as well as targeted LC–MS assays for the detection of a range of carnitines, aqueous metabolites and eicosanoids [1]. The carnitine, aqueous and eicosanoid datasets are available in raw data form through MetaboLights (MTBLS278, MTBLS303).

## 2 Experimental design, materials and methods

### 2.1 Study design

All animal studies were ethically reviewed and carried out in accordance with Animals (Scientific Procedures) Act 1986 and the GSK Policy on the Care, Welfare and Treatment of Animals. GW625019, a PPAR-pan activator was administered to male Sprague–Dawley rats (Crl:CD (SD) strain), 12 animals per group, by daily oral gavage at 30,100, 300, and 1000 mg/kg/day for 13 weeks. A separate satellite group of animals (6 per group) were kept for a 4 week treatment free period in the control, intermediate 2 (300 mg/kg/day) and high (1000 mg/kg/day) dose groups (Table 1).

All samples were analysed for total fatty acid, intact lipids, carnitine and aqueous metabolite content. Eicosanoids were measured from a subset of the samples including the control, intermediate 2 (300 mg/kg/day) and high (1000 mg/kg/day) dose groups.

### 2.2 Sampling

Tissue samples were collected following an overdose of anaesthetic (halothane Ph. Eur. Vapour). Samples of the liver were immediately removed, weighed, and sections snap-frozen in liquid nitrogen. Samples were maintained at −80 °C until further analysis.

### 2.3 Extraction of total fatty acids, intact lipids, carnitines and aqueous metabolites

Methanol: chloroform solution (2:1, 600 µL) was added to approximately 50 mg of frozen tissue and homogenised with a tissue lyser. Chloroform and water (200 µL each) was added, samples were sonicated for 15 min and centrifuged (13,500 rpm, 20 min). The resulting aqueous and organic layers were separated and the extraction procedure was repeated. Samples were dried under nitrogen and processed for mass spectrometry.

### 2.4 GC–MS analysis of fatty acid methyl esters (FAMEs)

Organic fractions were reconstituted in 1 mL of methanol:chloroform 2:1 and a fifth of each sample (200 µL) was dried under nitrogen. Chloroform:methanol (1:1, 100 μl), boron trifluoride in methanol (10%, 125 μl) and 150 µL D-25-tridecanoic acid (200 µM in chloroform) were added to the dried extracts. Samples were vortex mixed and heated to 80 °C for 90 min. After cooling, 300 µL water and 600 µL hexane were added, samples were vortex mixed, the lower aqueous layer was discarded and the remaining organic layer dried under nitrogen. The samples were reconstituted in 150 μl hexane and transferred to autosampler vials prior to analysis using a Trace GC Ultra coupled to a DSQ II single-quadrupole mass spectrometer (Thermo Scientific, Hemel Hempstead, Hertfordshire). Samples were injected onto a Zebron™ ZB-WAX column (100% polyethylene glycol 30 m×0.25 mm ID, 0.25 µm film thickness). The injector temperature was 230 °C and the flow rate of helium was 1.2 mL/min. The column was held at 60 °C for 2 min, after which the temperature was increased to 150 °C at a rate of 15 °C/min, and finally increased to 240 °C at a rate of 2.5 °C/min. The transfer line temperature was maintained at 240 °C, while the ion source was at 250 °C, operating at 70 eV for electron ionisation (EI). The detector was initiated after 240 s, and full scan spectra were collected over a range of 50–650 *m*/*z* [2], [3]. GC–MS chromatograms were processed using Xcalibur™ (version 2.0; Thermo Electron, Waltham, Massachusetts) (Supplementary Data 1).

### 2.5 Open profiling LC–MS/MS analysis of intact lipids

A 10 µL aliquot, comprising one hundredth of the organic fraction, was diluted into 90 µL of methanol-chloroform (2:1) containing 20 µM 1,2-diheptadecanoyl-sn-glycero-3-phosphocholine (PC (17:0/17:0)) (Avanti Polar Lipids Inc., Alabaster, Alabama, US) The instrumentation comprised a Xevo G2 Quadrupole Time of Flight (QToF) mass spectrometer with a Z-spray electrospray source (Waters Ltd., Elstree, Hertfordshire, UK) coupled to an ACQUITY Ultra Performance Liquid Chromatography (UPLC) system with an Acquity CSH C18, 1.7 µm (2.1×100 mm) column (Waters Ltd., Elstree, Hertfordshire, UK). Mobile phase A consisted of 10 mM ammonium formate in acetonitrile: water (6:4), whilst mobile phase B contained 10 mM ammonium formate in isopropanol: acetonitrile (9:1). The concentration of mobile phase B was increased from 40% to 100% over 18 min, then equilibrated to 40% B for 2 min at a flow rate of 0.4 mL/min. The electrospray source was operated in positive ion mode with the source temperature set at 80 °C and a cone gas flow of 100 L/h. The desolvation gas temperature was 250 °C and the nebuliser gas flow rate was set at 700 L/h. The capillary voltage was 3 kV and the cone voltage 50 V. Mass spectrometric data were collected from 50 to 1200 *m*/*z* in profiling scan mode. Data were processed using MarkerLynx™ within the software suite MassLynx™ (version 4.1) by Waters Ltd. (Elstree, Hertfordshire, UK). Collection Parameters were set with a mass window of 0.05 Da and retention time window of 0.2 min (Supplementary Data 2).

### 2.6 Analysis of acyl-carnitines

One hundred microliters of internal standard solution mix (1.63 µM [D9] free carnitine, 0.3 µM [D3] acetyl carnitine, 0.06 µM [D3] propionyl-carnitine, 0.06 µM [D3] butyryl-carnitine, 0.06 µM [D9] isovarelyl-carnitine, 0.06 µM [D3] octanoyl-carnitine, 0.06 µM [D9] myristoyl-carnitine, and 0.12 µM [D3] palmitoyl-carnitine, Cambridge Isotope Laboratories, Andover, MA, USA) was added to 40 µL of the organic fraction of the methanol: chloroform extraction and the resulting mixture were dried down under nitrogen and derivatised with 100 µL of 3 M butanolic-HCl (Sigma-Aldrich, Louis, Missouri, USA). Samples were evaporated under nitrogen, re-constituted and sonicated in 4:1 acetonitrile: 0.1% formic acid in water before transferring them to autosampler vials. Samples were analysed using an AB Sciex 5500 QTRAP mass spectrometer (AB Sciex UK Limited, Warrington, Cheshire) coupled to an Acquity UPLC system. Mobile phase A consisted of 0.1% formic acid in water, while mobile phase B was acetonitrile. Two microliters of each sample was injected onto a Synergi Polar RP phenyl ether column (100 mm×2.1 mm, 2.5 µm) supplied by Phenomenex (Macclesfield, Cheshire, UK). The analytical gradient started at 30% B, followed by a linear increase to 100% B over 3 min. The gradient was then held at 100% B for 5 min, after which it was returned to the re-equilibration level of 30% B for 2 min. A flow rate of 0.5 mL/min was used throughout [4]. Data were analysed using the Quantitation Wizard within Analyst™ version 1.6 by AB Sciex Ltd. (Warrington, Cheshire, UK) (Supplementary Data 3).

### 2.7 Targeted analysis of aqueous metabolites

The entire aqueous fraction was dissolved in 300 µl of 70:30 acetonitrile: water containing 20 µM universally ^13^C- and ^15^N- labelled glutamate. Samples were vortex mixed, sonicated, centrifuged, (17,000*g*, 5 min) pipetted into auto sampler vials and analysed using an AB Sciex 5500 Qtrap mass spectrometer (AB Sciex UK Limited, Warrington, Cheshire) coupled to a SIL20-A LC system (Shimadzu Corp., Kyoto, Japan). Mobile phase A consisted of 100 mM ammonium acetate, mobile phase B was acetonitrile, and the flow rate was 0.3 mL/min. Two microliters of each sample was injected, and analytes separated using a 100 mm ZIC-HILIC column with 2.1 mm ID and 3.5 µm particle size (Sequant, Umeå, Sweden). A linear gradient was used, starting at 20% A for 2 min, followed by an increase to 50% A over 10 min, and finally a 3 min re-equilibration. Metabolites of interest were measured in positive ionisation mode with unscheduled multiple reaction monitoring events (MRMs) (Table 2), using a source temperature of 500 °C, an ion spray voltage of 4.5 kV and a dwell time of 50 ms. Peaks were integrated by the Quantitation Wizard within Analyst™ version 1.6 by AB Sciex Ltd. (Warrington, Cheshire, UK) and normalised against wet tissue weight and to the intensity of the internal standards (Supplementary Data 4).

### 2.8 Extraction and analysis of eicosanoids

Eicosanoids were extracted using solid phase extraction (SPE) Waters Oasis-HLB cartridges (Waters Ltd., Elstree, Hertfordshire, UK) [4]. SPE columns were washed with ethyl acetate (2 mL), methanol (2×2 mL), and 15% methanol with 0.1% acetic acid (2 mL). Approximately 100 mg liver tissue samples were homogenised on a TissueLyser (Qiagen Ltd., Manchester, UK; 10 min at 30 Hz) in 1.5 mL 15% methanol with 0.1% acetic acid. The samples were centrifuged (17,000g, 2 min) and the supernatant loaded onto the SPE columns. Cartridges were washed with 1 mL 15% methanol with 0.1% acetic acid. Analytes of interest were eluted with 0.5 mL of methanol followed by 1 mL of ethyl acetate and immediately dried under nitrogen. Samples were finally reconstituted in 40 µL methanol containing 70 nM PGE2-d4 internal standard and transferred to autosampler vials. Analysis was performed using a 4000 QTRAP mass spectrometer (AB Sciex UK Limited, Warrington, Cheshire) coupled to an Acquity ultra performance liquid chromatography (UPLC) system (Waters Corp., Milford, MA). The autosampler was maintained at 4 °C, LC separation was achieved using a Luna, 3 μm particle size, 150×2 mm column (Phenomenex Macclesfield, Cheshire, UK). The gradient of mobile phase A (0.1% acetic acid in water) and B (0.1% acetic acid in acetonitrile: methanol 80:20) is detailed in Table 3. The flow rate was held at 0.4 mL/min. Metabolites of interest were measured in negative ionisation mode with unscheduled multiple reaction monitoring events (MRMs) (Table 4). Peaks were integrated by the Quantitation Wizard within Analyst™ version 1.6 by AB Sciex Ltd. (Warrington, Cheshire, UK) (Supplementary Data 5).

## Figures and Tables

**Table 1:** Study design.

Group description	Dose (mg/kg/day)	Animal number	Recovery animals
Control	0	1–12	13–18
Low	30	19–30	−
Intermediate 1	100	31–42	−
Intermediate 2	300	43–54	55–60
High	1000	61–72	73–78

**Table 2:** A list of mass to charge (*m/z*) ratios showing [M−H]^−^ values for each measured aqueous metabolite and the corresponding fragment ion after collision-induced dissociation (CID). Q1 denotes the parent ions, whereas Q3 denotes the fragment ions.

Analyte	Q1	Q3	Analyte	Q1	Q3
3-PG	187.0	105.0	GSH	308.1	179.0
Acetyl-CoA	810.0	303.2	GSSG	613.1	355.0
Adenine	136.0	119.0	GTP	523.9	152.0
Adenosine	268.1	136.1	Guanine	152.0	134.9
ADP	428.0	136.0	Guanosine	284.1	152.1
AMP	348.0	136.0	Malonyl-CoA	854.0	347.1
ATP	508.0	136.0	Methyl-cytosine	126.0	109.1
cAMP	330.1	136.1	NAD	664.0	427.9
CDP-choline	489.1	184.1	Oxo-methionine	165.0	105.0
cGMP	346.1	152.1	PCr	212.1	177.1
CMP	324.1	112.0	PEP	169.1	150.9
Cytidine	244.1	112.0	SAH	385.1	136.1
Cytosine	112.0	95.0	SAM	399.0	250.1
FAD	786.1	348.0	UMP	325.1	96.9
GDP	444.0	152.0	Uracil	112.9	70.1
GMP	364.2	152.1	Uridine	245.1	112.9

**Abbreviations:** 3PG, 3-phosphoglycerate; ADP, adenosine diphosphate; AMP, adenosine monophosphate; ATP, adenosine triphosphate; cAMP, cyclic adenosine monophosphate; cGMP, cyclic guanosine monophosphate; CMP, cytidine monophosphate; FAD, flavin adenine dinucleotide; GDP, guanosine diphosphate; GMP, guanosine monophosphate; GSH, glutathione; GSSG, oxidised glutathione; GTP, guanosine triphosphate; NAD, nicotineamide adenine dinucleotide; PCr, phosphocreatine; PEP, phosphoenolpyruvate; SAH, S-adenosyl-homocysteine; SAM, S-adenosyl-methionine; UMP, uridine monophosphate.

**Table 3:** Eicosanoid method gradient.

Analysis		
Column	C_18_ Luna column (Phenomenex)	
column dimensions	150 mm×2 mm, 3 µm	
Liquid chromatograph	Waters Acquity	
flow rate	0.4 mL/min	
mobile phase A	0.1% acetic acid	
mobile phase B	0.1% acetic acid	
	in acetonitrile: methanol (80:20)	
Gradient	0 min	15% B
	1.5 min	30% B
	10.5 min	60% B
	16 min	80% B
	19 min	100% B
	19.1 min	15% B
	21 min	15% B

**Table 4:** A list of mass to charge (*m/z*) ratios showing [M−H]^−^ values for each measured eicosanoid and the corresponding fragment ion after collision-induced dissociation (CID). Q1 denotes the parent ions, whereas Q3 denotes the fragment ions.

Analyte	Q1	Q3	Analyte	Q1	Q3
11(12)-EET	319.2	167	8,9-DHET	337.2	127.1
11,12,15-THET	353.2	167.1	8-HETE	319.2	301.2
11,12-DHET	337.2	167.1	8-iso-PGE_2_	351	271
11-HEPE	317	169	8-isoPGF_2_α	353.1	193.2
11-HETE	319.18	166.9	9(10)-EpOME	295.2	171.1
12(13)-EpOME	295.2	195.2	9,10,13-TriHOME	329.2	171.1
12,13-DHOME	313.2	183.2	9,10-DHOME	313.2	201.2
12-HEPE	317.17	179	9,12,13-TriHOME	329.2	211.1
12-HETE	319.2	179.2	9-HODE	295.2	171
13-HDoHE	343.13	193	9-oxo-ODE	292.2	185.1
13-HODE	295.2	195	AA	303.3	259.1
13-oxo-ODE	239.2	113	DHEA	327.2	283.1
14(15)-EET	319.2	219.3	DGLA	305.19	58.8
14,15-DHET	337.19	206.9	Lipoxin A4	351.2	115.2
15-deoxyPGJ2	315.2	271.3	LTB_4_	335.2	195.1
15-HETE	319.2	301.4	PGB_2_	333.069	59
15-oxo-EET	317.2	113.1	PGD_2_	351.2	271.3
19-HETE	319.2	275.1	PGE_2_	351.2	271.3
20-HETE	319.2	275.2	PGE_2_-d_4_	355.3	275.3
5(6)-EET	319.2	191	PGF_2_α	353.2	309.3
5,6-DHET	337.2	145.1	THF diols	353.2	167.1
5-oxo-EET	317.2	273.2	TXB_2_	369.2	169.1

**Abbreviations**: AA, arachidonic acid; DGLA, dihomo-γ-linolenic acid; DHEA, docosahexaenoic acid; DHET, dihydroxyeicosatrienoic acid; DHOME, dihydroxyoctadecenoic acid; EET, epoxyeicosatrienoic acid; HDoHE, hydroxydocosahexaenoic acid; HEPE, hydroxyeicosapentaenoic acid; HETE, hydroxyeicosatetraenoic acid; HODE, hydroxyoctadienoic acid; LT, leukotriene; ODE, octadienoic acid, PG, prostaglandin; THET, trihydroxyeicosatetraenoic acid; THF, tetrahydrofuran; TX, thromboxane.

## Appendix A Supplementary material

Supplementary material

Supplementary material:**Supplementary Data 1:** GC–MS dataset on the analysis of fatty acid methyl esters (FAMEs) as measured using a Trace GC Ultra coupled to a DSQ II mass spectrometer. Lipids were measured from the extracts of liver tissue samples treated with PPAR-pan agonist GW625019. Metabolite IDs were verified using mass fragmentation patterns matched to the National Institute of Standards and Technology (NIST) library, and supported by retention time and fragment comparison with a FAME mix of 37 compounds. The intensity of the analytes were normalised to the intensity of the internal standards.

Supplementary material:**Supplementary Data 2:** LC–MS dataset of intact lipids as measured on a Waters Xevo G2 (Q-ToF) mass spectrometer coupled to an Acquity UPLC system. Lipid were measured from the extracts of liver tissue samples treated with PPAR-pan agonist GW625019. Area ratio values are listed, for each mass to charge and retention time pairs where the intensity of the selected features are normalised to the intensity of the internal standard used.

Supplementary material:**Supplementary Data 3:** LC–MS of acyl-carnitines measured on an AB Sciex 5500 Qtrap mass spectrometer coupled to an Acquity UPLC system. Carnitines were measured from the organic extracts of liver tissue samples treated with PPAR-pan agonist GW625019. Common product ions at *m/z* 85 was used to identify acylcarnitine [M+H]^+^ ions. The intensity of the analytes were normalised to the intensity of the internal standards.

Supplementary material:**Supplementary Data 4:** LC–MS of aqueous metabolites as measured using an AB Sciex 5500 Qtrap mass spectrometer coupled to a SIL20-A LC system. Authentic standards were used for the development and the identification of the listed analytes. Peak areas were normalised to the peak areas of the internal standards.

Supplementary material:**Supplementary Data 5:** LC–MS of eicosanoid metabolites measured on an AB Sciex 4000 Qtrap mass spectrometer coupled to an Acquity UPLC system. Authentic standards were used for the development and the identification of the listed analytes. Peak areas were normalised to the peak areas of the internal standards.

## Footnotes

Appendix A Supplementary data associated with this article can be found in the online version at doi:10.1016/j.dib.2016.05.002.
