# Supplementary material for: Metabolomics dataset of PPAR-pan treated rat liver
Source: Data Brief. 2016 May 10;8:196–202. doi: 10.1016/j.dib.2016.05.002 (PMC4898904; doi:10.1016/j.dib.2016.05.002)
Supplement: Supplementary file 1 — Supplementary material [file mmc1.docx]

Declaration of Conflicts of Interest

A.W.N. declares he is an employee of GlaxoSmithKline who part funded the project. All other authors declare they have no direct conflict of interest.
